# Rhamnetin, a Natural Flavonoid, Ameliorates Organ Damage in a Mouse Model of Carbapenem-Resistant *Acinetobacter baumannii*-Induced Sepsis

**DOI:** 10.3390/ijms232112895

**Published:** 2022-10-25

**Authors:** Hyeju Lee, Manigandan Krishnan, Minju Kim, Young Kyung Yoon, Yangmee Kim

**Affiliations:** 1 Department of Bioscience and Biotechnology, Konkuk University, Seoul 05029, Korea; 2 Division of Infectious Diseases, Department of Internal Medicine, College of Medicine, Korea University Anam Hospital, Korea University, Seoul 02841, Korea

**Keywords:** sepsis, rhamnetin, lung recovery, *Escherichia coli*, carbapenem-resistant *Acinetobacter baumannii*

## Abstract

In sepsis, the persistence of uncontrolled inflammatory response of infected host cells eventually leads to severe lung and organ failure and, ultimately, death. Carbapenem-resistant *Acinetobacter baumannii* (CRAB), causative bacteria of sepsis and lung failure in acute cases, belongs to a group of critical pathogens that cannot be eradicated using the currently available antibiotics. This underlines the necessity of developing new modes of therapeutics that can control sepsis at the initial stages. In this study, we investigated the anti-inflammatory activities in vitro and in vivo and the antiseptic effects of rhamnetin, a naturally occurring flavonoid. We found that among its isoforms, the potency of rhamnetin was less explored but rhamnetin possessed superior anti-inflammatory activity with least cytotoxicity. Rhamnetin showed significant anti-inflammatory effects in lipopolysaccharide-, CRAB-, and *Escherichia coli* (*E. coli*)-stimulated mouse macrophages by inhibiting the release of interleukin-6 and nitric oxide. In a mouse model of sepsis infected with clinically isolated CRAB or *E. coli*, rhamnetin significantly reduced the bacterial burden in the organs. In addition, normalized pro-inflammatory cytokine levels in lung lysates and histological analysis of lung tissue indicated alleviation of lung damage. This study implies that a potent natural product such as rhamnetin could be a future therapeutic for treating carbapenem-resistant gram-negative sepsis.

## 1. Introduction

Sepsis is a serious disease that is the leading cause of death due to infection worldwide and has hence been classified as a cause of global health concern [1]. Gram-negative bacteria affect hosts by producing various virulence factors. Lipopolysaccharide (LPS), an endotoxin released from the outer membrane of gram-negative bacteria, triggers the initiation of the Toll-like receptor (TLR) signaling cascade [2]. This can cause a systemic inflammatory response, leading to organ dysfunction or sepsis due to uncontrolled local inflammation by gram-negative bacteria in the infected tissues [3]. Despite the steady development of advanced antibiotics, the mortality rate due to sepsis caused by gram-negative bacteria is high. Hence, research on new types of therapeutics is required to expand the treatment options.

Antibiotic-resistant gram-negative pathogens are causes of global health concern [4]. Carbapenem, such as imipenem and meropenem, are effective antibiotics for treating gram-negative bacterial infections; however, bacterial resistance to carbapenem has become widespread because of excessive drug use [5]. Carbapenem-resistant *Acinetobacter baumannii* (CRAB) belongs to a group of critical pathogens for which the World Health Organization (WHO) declared an urgency to develop new antibiotics in 2017 [6]. However, treatment options are still limited, as time is required for properly optimizing antibiotic doses and assessing toxicities [7]. Therefore, new antibiotics and future perspectives for curing sepsis are urgently required. Many patients with sepsis are at risk of developing acute lung damage, which is associated with mortality due to sepsis [8,9]. The permeability of lung endothelial cells increases due to the induction of cytokine storms during sepsis, leading to pulmonary edema [10]. This progressive lung injury causes acute lung injury (ALI) or acute respiratory distress syndrome (ARDS), which is characterized by a rapid onset of respiratory failure [11]. However, effective drugs for these diseases, especially for antibiotic-resistant gram-negative infections, are lacking. This suggests that pre-blocking the excessive inflammatory response is one way of preventing lung damage [12].

Early administration of appropriate treatment is important for treating sepsis. However, with the rapid increase in antibiotic resistance, mortality in sepsis-related patients is also increasing because of failure of the initial lines of treatment [13]. Hence, supplementing existing drugs with new therapeutics appears to be a viable approach for countering drug resistance. Plant-based natural agents are good candidates, as the search for naturally occurring compounds that can inhibit the TLR4 activation pathway is underway [14]. Natural compounds are generally less toxic than chemical compounds and are readily available in food. Secondary metabolites of plants are valuable resources; in particular, studies on their pharmacological properties are actively pursued [15].

**Figure 1:**
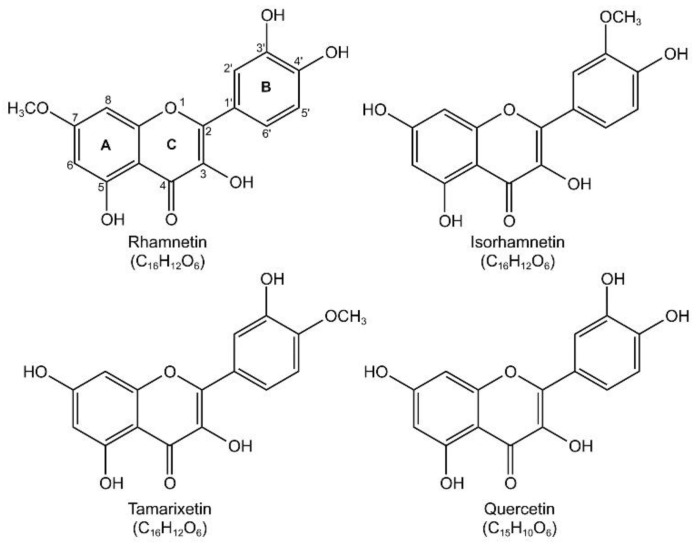
Chemical structure of rhamnetin (2-(3,4-dihydroxyphenyl)-3,5-dihydroxy-7-methoxychromen-4-one) and its isoforms (isorhamnetin (3,5,7-trihydroxy-2-(4-hydroxy-3-methoxyphenyl)chromen-4-one), tamarixetin (3,5,7-trihydroxy-2-(3-hydroxy-4-methoxyphenyl)chromen-4-one), and quercetin (2-(3,4-dihydroxyphenyl)-3,5,7-trihydroxychromen-4-one)). The image was drawn using KingDraw software (http://www.kingdraw.cn/, accessed on 21 August 2022).

Polyphenols constitute a large portion of a group of plant secondary metabolites [16]. Flavonoids, a group of plant pigments with a polyphenol-flavan backbone, possess a broad range of biological activities [17,18] and have been studied extensively for their antioxidant, anti-inflammatory, antibacterial, antiviral, and anti-allergic effects and are attracting attention as new candidates for the treatment of infectious diseases [19,20,21]. Many flavonoids have been reported to exhibit anti-inflammatory activity [22]. In particular, we reported that O-methylated flavonols, such as isorhamnetin [23] and tamarixetin [24], can reduce inflammatory responses (Figure 1).

Rhamnetin is a flavonoid derived from *Rhamnus petiolaris*, *Coriandrum sativum*, *Syzygium aromaticum*, and *Prunus cerasus* [25] and contains a methoxy group in ring A that provides better metabolic stability and pharmacological effects than non-methoxylated quercetin [15]. Methylation renders the structure sterically bulky, alters coplanarity, and reduces antioxidant capacity [26]. However, O-methylation tends to improve bioavailability [27]. Isorhamnetin is O-methylated at position 3′ of ring B, and rhamnetin is O-methylated at position 7 of ring A; the O-methylation can result in differences in receptor interaction. It has been reported that rhamnetin isoforms, isorhamnetin and tamarixetin at doses of 1 mg/kg and quercetin at doses up to 100 mg/kg, have significant effects on the infection induced by *Escherichia coli* (*E. coli*) [23,24,28]. In addition, the antibacterial effect of rhamnetin on *Mycobacterium tuberculosis* was confirmed, along with anti-inflammatory activities in MRC-5 human lung fibroblasts [29]. We found that compared to other O-methoxy flavonols, such as isorhamnetin and tamarixetin, rhamnetin is less toxic to mammalian cells [30].

In this study, we investigated the anti-inflammatory activity of rhamnetin against gram-negative bacterial infections in vitro and in vivo. In particular, we examined the potency of rhamnetin in inducing recovery from lung damage caused by gram-negative sepsis. This study provides insights regarding the potential of rhamnetin, a natural product, as a therapeutic for curing CRAB-induced sepsis.

## 2. Results

### 2.1. Rhamnetin Inhibits Several Inflammatory Markers

**Figure 2:**
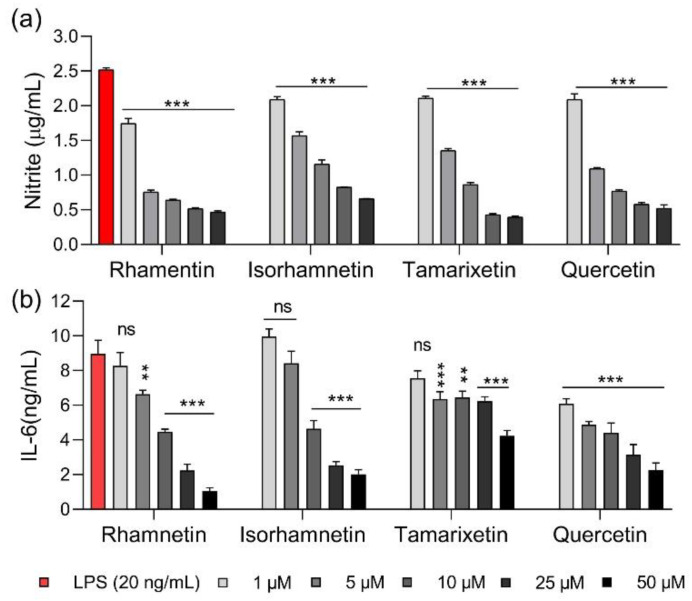
Anti-inflammatory activities of rhamnetin and its isoforms. (**a**) Inhibition of nitric oxide production by flavonoids in lipopolysaccharide (LPS)-stimulated RAW 264.7 cells. (**b**) Inhibition of interleukin 6 (IL-6) production by flavonoids in LPS-treated RAW 264.7 cells. Data are presented as mean ± SEM from triplicate experiments. ** *p* < 0.01; *** *p* < 0.001; and ns, nonsignificant compared to that in the LPS group.

Natural products, such as flavonols, have been reported to suppress the production of pro-inflammatory substances in LPS-stimulated macrophages [31]. Here, we measured the anti-inflammatory activities of four flavonol isoforms, namely quercetin, rhamnetin, isorhamnetin, and tamarixetin. Nitric oxide (NO) is an inflammatory mediator that induces an inflammatory response when overproduced. NO production increased in LPS-stimulated RAW 264.7 cells stimulated with LPS, as shown in Figure 2a. Flavonoids were added at concentrations ranging from 1 to 50 μM. In particular, rhamnetin inhibited NO production by 36.1% at 1 μM and by approximately 95.7% at 50 μM, showing that its NO-inhibiting activities were better than those of other flavonols. Production of interleukin 6 (IL-6), a pro-inflammatory cytokine secreted primarily by monocytes, was inhibited by 76.3%, 52.0%, and 73.8% when the cells were treated with 50 μM isorhamnetin, tamarixetin, and quercetin, respectively, while rhamnetin showed a slightly higher inhibition rate at all concentrations than the other flavonols (Figure 2b).

### 2.2. Rhamnetin Is Less Toxic Than Its Isoforms to Mammalian Cells

**Figure 3:**
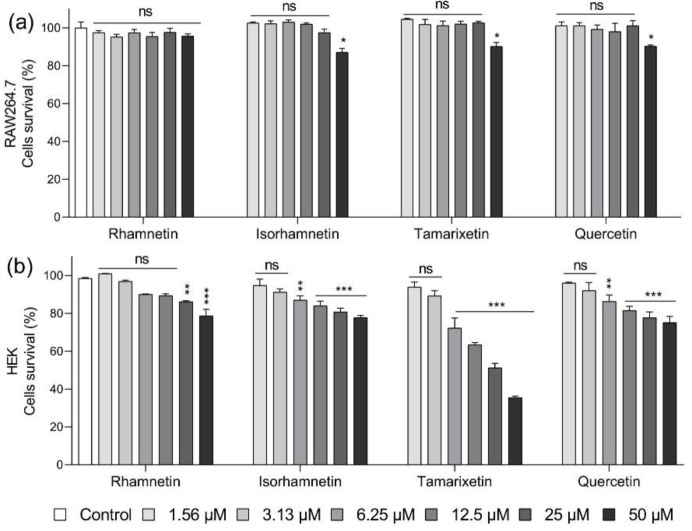
In vitro cytotoxicities of rhamnetin and its isoforms in (**a**) RAW 264.7 cells and (**b**) human embryonic kidney (HEK) cells. Data are presented as mean ± SEM from triplicate experiments. * *p* < 0.05, ** *p* < 0.01; *** *p* < 0.001; and ns, nonsignificant compared to that in the control group.

To confirm the safety of flavonoids, we treated each cell line with a range of different concentrations (maximum concentration being 50 μM) of the four flavonoids and measured their toxicity. Viability was calculated based on the number of cells cultured in cell culture medium containing 0.16% DMSO without flavonoid treatment. DMSO was added based on the application of 50 μM flavonoid. As shown in Figure 3a, 95.7% of the RAW 264.7 cells were viable after treatment with even 50 μM rhamnetin, while the same concentration of other flavonoids decreased the survival rate to approximately 10%. In human embryonic kidney (HEK) cells, rhamnetin at 25 and 50 μM maintained a survival rate of more than 86% and 78%, respectively. In contrast, cell viability was 80.8% and 77.8% when treated with 25 and 50 μM isorhamnetin, respectively; 51.3% and 35.4% with 25 and 50 μM tamarixetin, respectively; and 77. 8% and 75.1% with 25 and 50 μM quercetin, respectively (Figure 3b). These findings confirm that rhamnetin was less toxic than its isoforms. These results showed that mammalian cells could tolerate rhamnetin better than isorhamnetin, as previously confirmed in NIH3T3 mouse embryonic fibroblasts and MRC-5 cells [29].

### 2.3. Rhamnetin Inhibits the Production of Cytokines in E. coli-Infected Mouse Macrophages

**Figure 4:**
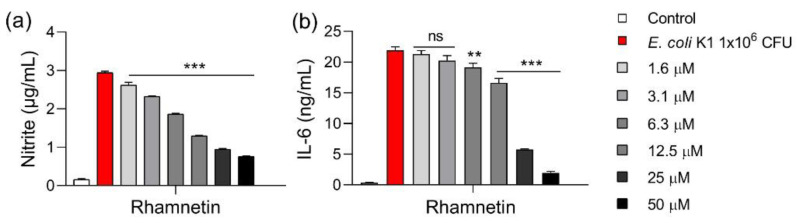
Effect of rhamnetin on *Escherichia coli*-induced (**a**) nitrite and (**b**) IL-6 productions in RAW 264.7 cells. The cells were stimulated with 1 × 10^6^ colony forming unit (CFU) of *E. coli* at 37 °C for 16 h. Data are presented as mean ± SEM from triplicate experiments. ** *p* < 0.01; *** *p* < 0.001; and ns, nonsignificant compared to that in the *E. coli* group.

For further investigation, we selected rhamnetin as a therapeutic candidate because of its potent anti-inflammatory activity and low toxicity in mammalian cells compared to its isoforms. We investigated whether rhamnetin maintains anti-inflammatory activity in cells not only stimulated by LPS released from the outer membrane of gram-negative *E. coli* but also by pathogenic bacteria that actually cause infections. The RAW 264.7 cells were stimulated by *E. coli* and the level of inflammatory markers was measured. NO production was suppressed by 11.6% and 78.2% in response to 1.6 μM and 50 μM rhamnetin, respectively (Figure 4a). IL-6 production was inhibited by 12.9% and 92.5% in response to 6.3 μM and 50 μM rhamnetin, respectively. Therefore, rhamnetin showed anti-inflammatory activity in cells stimulated with *E. coli* and LPS (Figure 4b).

### 2.4. Rhamnetin Inhibits Cytokine Production in CRAB-Infected Mouse Macrophages

**Figure 5:**
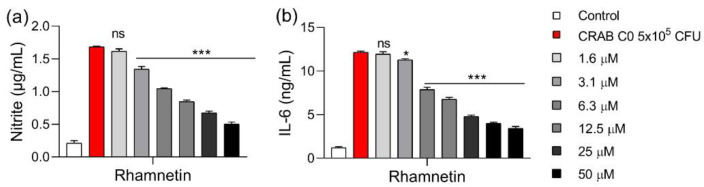
Effect of rhamnetin on (**a**) nitrite and (**b**) IL-6 levels in RAW 264.7 cells stimulated with carbapenem-resistant *Acinetobacter baumannii* (CRAB) C0. The cells were stimulated with 5 × 10^5^ CFU of CRAB at 37 °C for 16 h. Data are presented as mean ± SEM from triplicate experiments. * *p* < 0.05; *** *p* < 0.001; and ns, nonsignificant compared to that in the CRAB group.

We investigated the anti-inflammatory activity of rhamnetin in macrophages stimulated with clinically isolated CRAB C0. In these cells, 3.1 μM rhamnetin inhibited NO production by 22.3%; the inhibition rate increased to 77.5% in the presence of 50 μM rhamnetin (Figure 5a). IL-6 production also decreased with increasing concentrations of rhamnetin; 3.1 μM rhamnetin inhibited IL-6 production by 5.4%, 12.5 μM by 49.1%, and 50 μM by 74.4% (Figure 5b). Therefore, these results confirmed that rhamnetin could sufficiently inhibit cytokine production, even in CRAB-stimulated cells.

### 2.5. Rhamnetin Treatment Is Effective for E. coli-Induced Sepsis

**Figure 6:**
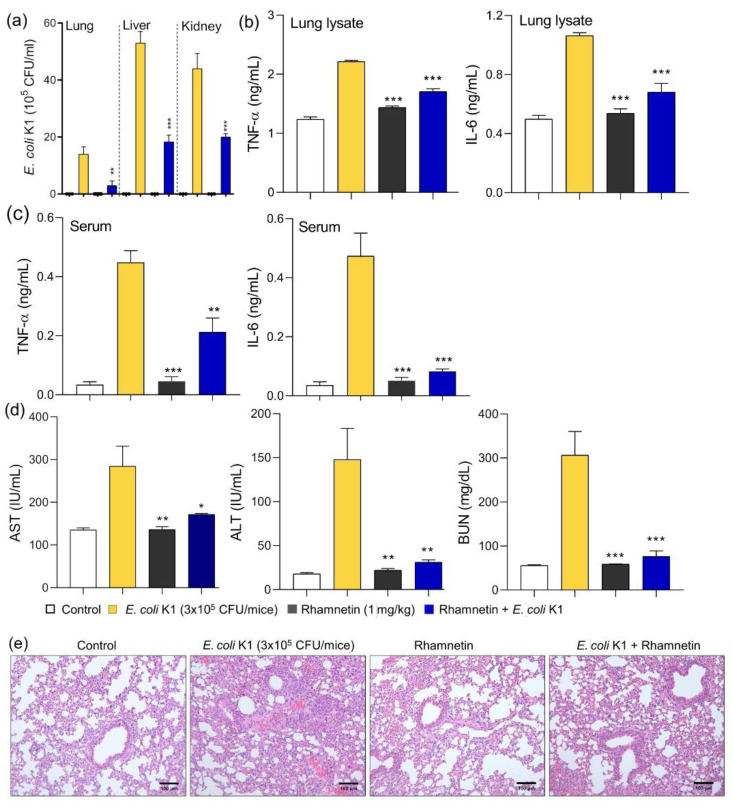
Effects of rhamnetin on *E. coli*-induced sepsis. (**a**) Evaluation of bacterial load in the visceral organs of mice. Cytokine (TNF-α and IL-6) levels (**b**) in the lung lysates and (**c**) in the serum. (**d**) Aspartate aminotransferase (AST), alanine aminotransferase (ALT), and blood urea nitrogen (BUN) levels in the serum. (**e**) Hematoxylin and eosin staining of the lung tissue in a model of *E. coli*-induced sepsis. Images are at 20× magnification and scale bar is 100 μm. Data are presented as mean ± SEM from triplicate experiments. * *p* < 0.05; ** *p* < 0.01; *** *p* < 0.001 compared to that in the *E. coli* group.

Studies have shown that isorhamnetin, tamarixetin, and quercetin suppress systemic infection in mouse models of *E. coli*-induced sepsis or endotoxemia [23,24,28]. In this study, we evaluated the antiseptic activity of rhamnetin in an *E. coli*-induced mouse model of sepsis. 1 mg/kg of rhamnetin was injected 1 h before treating the mice with 3 × 10^5^ colony forming unit (CFU) of *E. coli,* and the efficacy of rhamnetin was analyzed after 16 h. 1 mg/kg concentration was selected based on our previous studies [23,24,32,33]. Rhamnetin inhibited bacterial growth in vivo, because of which the number of bacteria in the lung, liver, and kidney reduced significantly in the rhamnetin-treated group (Figure 6a). As shown in Figure 6b, cytokine secretion increased in the *E. coli*-treated group; however, the cytokine level decreased in the group injected with rhamnetin. In the lung lysate, TNF-α and IL-6 levels decreased by 51.8% and 67.7%, respectively, after rhamnetin treatment. In addition, the amount of cytokine secretion in serum was also analyzed. TNF-α was inhibited by 56.9% and IL-6 inhibition was 89.4% by rhamnetin (Figure 6c). The levels of organ damage markers, such as aspartate transaminase (AST), alanine aminotransferase (ALT), and blood urea nitrogen (BUN), were analyzed to evaluate organ dysfunction due to sepsis. A group of mice with *E. coli*-induced sepsis showed increased levels of these markers due to liver and kidney damage (Figure 6d). However, organ damage was prevented in the group treated with rhamnetin, and the levels of the markers decreased significantly below the statistical *p*-value of 0.05. In addition, the organ damage marker levels did not increase, even in the group treated with rhamnetin, implying that rhamnetin is not toxic for the liver and kidney. To investigate the inflammatory response in the lungs, the lung tissue was observed under a microscope after hematoxylin and eosin staining. Severe edema and alveolar hemorrhage were observed in lung tissues infected with *E. coli*. However, the lung tissue damage was alleviated in the group pretreated with rhamnetin (Figure 6e). This suggested that rhamnetin inhibited the inflammatory response in an *E. coli*-induced model of septic shock.

### 2.6. Rhamnetin Protects Mice from CRAB-Induced Septic Shock

**Figure 7:**
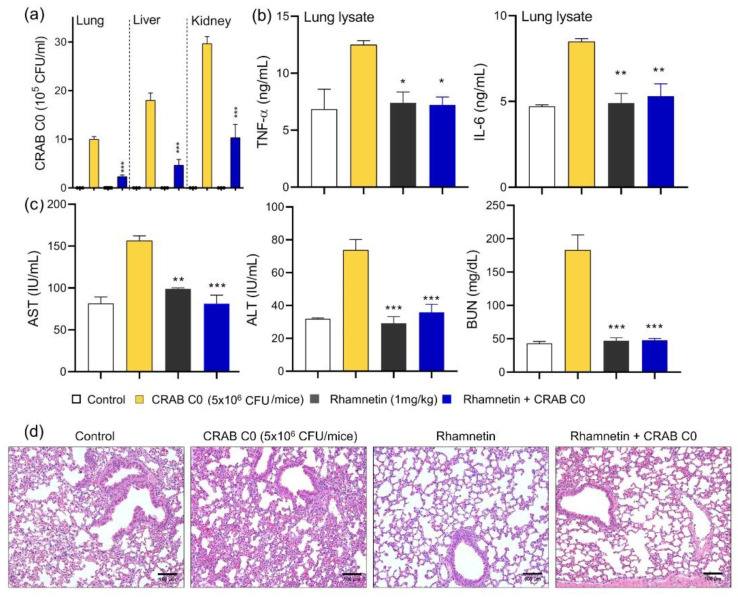
Effects of rhamnetin on CRAB C0-induced sepsis. (**a**) Evaluation of bacterial load in the visceral organs of mice. (**b**) Production of cytokines (TNF-α and IL-6) in the lung lysates. (**c**) AST, ALT, and BUN levels in the serum. (**d**) Hematoxylin and eosin staining of the lung tissue in a model of CRAB-induced sepsis. Images are at 20× magnification and scale bar is 100 μm. Data are presented as mean ± SEM from triplicate experiments. * *p* < 0.05; ** *p* < 0.01; *** *p* < 0.001 compared to that in the CRAB group.

Based on the anti-inflammatory activities of rhamnetin in CRAB-stimulated macrophages, we investigated for the first time the in vivo therapeutic potential of natural rhamnetin in a mouse model of clinically isolated CRAB C0-induced sepsis. To test whether rhamnetin inhibited CRAB proliferation in vivo, we examined the bacterial counts in the organs of mice with CRAB-induced sepsis. When rhamnetin (1 mg/kg) was injected 1 h before bacterial infection, the number of bacteria in the lungs, liver, and kidneys reduced significantly by 76.7%, 74.1%, and 65.2%, respectively, compared to that observed in the CRAB-injected group without rhamnetin treatment (Figure 7a), confirming the potential antibacterial ability of rhamnetin against CRAB in vivo. The reduction in cytokine levels was analyzed in the CRAB-induced mouse model of septic shock. The levels of inflammatory cytokines, TNF-α and IL-6, were upregulated in infected mice compared to those in normal mice. Rhamnetin injection in the mouse model of sepsis downregulated TNF-α by 93.2% and IL-6 by 84.5% (Figure 7b). The AST, ALT, and BUN levels confirmed that rhamnetin effectively protected the liver and kidney against CRAB infection. The AST and ALT levels in the experimental group treated with rhamnetin after infection with CRAB were almost identical to those in the control group injected with phosphate-buffered saline (PBS). BUN level was reduced by 96.8% (Figure 7c). Histological analysis of the lungs infected with CRAB confirmed alleviation of lung injury by rhamnetin. Compared to the control group, inflammatory cell recruitment and edema were observed in CRAB-infected mice. These changes were not observed in the group injected with rhamnetin, indicating that rhamnetin cured lung infection due to sepsis (Figure 7d).

## 3. Discussion

In this study, we demonstrated the antiseptic activity and therapeutic potential of rhamnetin in gram-negative sepsis. Rhamnetin exhibited the lowest toxicity and highest anti-inflammatory activity among its isoforms. Therefore, we selected rhamnetin as a candidate antibiotic for treating sepsis and confirmed its protective effect in CRAB- or *E. coli*-infected mouse models.

The host activates the innate and adaptive immune systems by mediating pro-inflammatory cytokines [34]. However, as the over-activation of pro-inflammatory cytokines leads to multi-organ failure, the initial anti-inflammatory function of macrophages plays a critical role in countering infections [35]. Hence, we measured the anti-inflammatory activity of rhamnetin after infecting murine macrophages with bacteria that continuously release various pathogen-associated molecular patterns, including LPS. Components of the immune system interact with each other in vivo; hence, animal models provide mechanistic insights beyond cellular experiments [36]. However, the data obtained from this type of analysis must be carefully analyzed because of the biological differences between humans and mice. Nevertheless, as new therapeutic agents cannot be tested in humans, mouse models provide the basis for new discoveries [37]. Hence, in this study, we have also used a mouse model of sepsis to analyze the anti-inflammatory activity of rhamnetin.

Beta-lactam antibiotics, including carbapenems, are typically used against gram-negative bacteria. However, the number of bacteria resistant to these beta-lactams is increasing, posing a threat to global health [38]. Therefore, the utility of antibiotics in treating carbapenem-resistant bacteria has become limited. Several antibiotics are currently administered to treat sepsis caused by carbapenem-resistant bacteria. Cefiderocol, a cephalosporin with siderophore properties, is effective in inhibiting cell membrane synthesis [39]. Tigecycline is a glycylcycline antibiotic and is a derivative of the tetracycline antibiotic class. It has broad antibacterial activity against multidrug-resistant bacteria [40,41]. Sulbactam is a beta-lactamase inhibitor that is not affected by OXA-23 and is clinically used due to its activity against CRAB [42]. In clinical practice, combination therapy is preferred over monotherapy [43]. In particular, colistin is recommended for use in combination therapy because it increases cases of colistin resistance [44]. However, colistin should be used cautiously because of its nephrotoxic and neurotoxic effects [44,45]. Therefore, new therapeutic agents that can replace traditional antibiotic therapies are urgently required. For example, it was reported that the soluble bacteriophage SH-Ab15519 effectively ameliorated lung infection with a long half-life in the target organ after intranasal administration to CRAB-infected mice [46]. Peptide therapy offers a novel solution. For instance, R-Pro9-3D, a 9-residue peptide derived from protaetiamycin, exhibits antibacterial and anti-inflammatory activities both in vivo and in vitro against CRAB, demonstrating its effectiveness as an antiseptic peptide [32]. Additionally, a novel antimicrobial peptide, PapMA-3, depolarized the CRAB membrane and acted synergistically with carbapenem antibiotics [33].

The anti-inflammatory action of flavonoids in LPS-stimulated RAW 264.7 cells has been extensively investigated [47]. Previous studies have shown that quercetin inhibits the activation of the phosphorylated tyrosine motif of myeloid differentiation primary-response protein 88 (MyD88), thereby inhibiting the activation of molecules acting downstream of MyD88 and limiting LPS-induced inflammatory mediators [48]. We previously demonstrated that rhamnetin binds to c-Jun N-terminal kinase (JNK) and p38 MAPK via extensive hydrogen bonding and additional hydrophobic interaction with high binding affinity. Therefore, downregulating of the phosphorylation of the MAPK signaling pathway by rhamnetin can inhibit overproduction of pro-inflammatory cytokines which is the leading cause of cytokine storm and death in sepsis patients [49]. We have also reported the anti-inflammatory mechanism of rhamnetin in IFN-γ-stimulated human lung fibroblast MRC-5 cells. Rhamnetin inhibits p38 and ERK activation and reduces the expression levels of matrix metalloproteinase (MMP)-1, IL-1β, IL-12, and TNF-α, thereby suppressing inflammation via MAPK signaling associated with mycobacterial infection [29]. Therefore, in this study, we used RAW 264.7 cells infected with sepsis-causing *E. coli* or CRAB, showing that rhamnetin reduced the production of inflammatory cytokines induced by gram-negative bacteria. This suggests that rhamnetin treatment protected macrophages from endotoxin- or virulent *E. coli*-induced inflammatory responses, as well as CRAB-induced inflammation.

Natural products are a good source of new antibiotics with low toxicity [50]. As antibacterial agents, it has been reported that some flavonoids promote phagocytosis of macrophages to eliminate pathogens [51,52]. The bacteriostatic effect of quercetin has been demonstrated to be because of the efflux of intracellular components through damaged bacterial cell membranes [53]. It was also demonstrated to down-regulate genes involved in cell-cell adhesion in biofilm-forming pathogens and contribute to the removal of virulence factors through antibiofilm activity [54]. Many studies have shown that various flavonoids can act as therapeutic agents for bacterial infection. Active fractions containing various flavonoids isolated from *Lolium multiflorum* are known for their anti-inflammatory activities and improvement of survival time in mice with septic shock [55]. Curcumin and licochalcone B inhibit the activation of the NLR family pyrin domain-containing protein 3 (NLRP3) inflammasome, thereby showing a protective effect against sepsis [56,57]. Reports have shown that quercetin protects the lungs from damage caused by oxidative stress in a rat model of sepsis by increasing the levels of antioxidant enzymes [58]. Treatment with rutin markedly reduces the amount of pro-inflammatory cytokines, protecting LPS-infected mice from excessive inflammatory responses [59].

Gram-negative bacterial infections can cause uncontrolled LPS-induced inflammatory signaling, resulting in acute sepsis. Sepsis induced by multidrug-resistant (MDR) gram-negative bacteria, such as CRAB, is a serious clinical problem that is difficult to treat, leading to high mortality rates. However, developing any therapeutic agent is challenging because of the worldwide transmission and modification of the carbapenem hydrolase oxacillinase gene, which is the main mechanism underlying carbapenem resistance [60]. Currently, proper treatment methods for sepsis-associated ARDS are lacking, and most patients are supported by mechanical ventilation [61]. Unfortunately, as this assistive device exerts detrimental effects on patient health and increases mortality [62], developing a fundamental mode of treatment is critical. To resolve these problems, we investigated the therapeutic potential of rhamnetin in ameliorating lung damage caused by *E. coli* and CRAB in a murine model of septic shock. Rhamnetin significantly reduced the number of bacteria in the lungs, liver, and kidneys of mice infected with *E. coli* and clinically isolated CRAB C0, which confirmed the alleviation of organ damage from bacterial infection. In particular, the blockade of sepsis progression that could have led to lung failure was confirmed visually. In sepsis-induced mouse lungs, neutrophil infiltration, edema, and hemorrhage were observed due to the disruption of the alveolar and capillary barriers. However, the rhamnetin-treated group did not show any increase in permeability and thus did not show edema or inflammation, similar to that observed in the untreated control group. This indicated that rhamnetin reduced lung damage. TNF-α and IL-6 participate in acute inflammatory responses that result in lung injury. Hence, reducing the production of these inflammatory cytokines in the lungs may protect the lungs from damage [63]. We confirmed the reduction in cytokine levels in the lung lysates in the rhamnetin-treated group; therefore, rhamnetin can be considered a prophylactic factor for acute lung inflammation. Interestingly, tamarixetin protected mice from sepsis by stimulating the secretion of the IL-10 [24]. IL-10 is known as an anti-inflammatory cytokine, limits inflammatory responses and involves in immunoregulation [64]. In contrast, isorhamnetin is known to inhibit TNF-α and IL-6 in a model of *E. coli*-induced sepsis [23]. In this study, we found that rhamnetin inhibits the production of TNF-α and IL-6 similar to isorhamnetin [23]. These results implied that the difference in O-methylation position of its isoforms may play important roles in interaction with target receptors in inflammatory signaling pathways. Further investigation of the underlying mechanisms will broaden the range of applications for rhamnetin.

Overall, this study is the first to show that the natural flavonoid, rhamnetin, protects mice from CRAB-induced sepsis and provides insights regarding the development of novel therapeutic candidates that can be used for preventing or treating gram-negative sepsis. Further studies are required to investigate the mechanism underlying its antiseptic activity and confirm the potency of rhamnetin as a new drug against antibiotic resistance.

## 4. Materials and Methods

### 4.1. Bacterial Strains

*E. coli* K1 strain (O18ac:K1:H7) was acquired from the American Type Culture Collection (ATCC 700973). This strain is highly toxic because it contains the K1 capsule, a polysaccharide virulence factor that interferes with host defense [65]. CRAB C0 is a carbapenem-resistant *A. baumannii* strain obtained from the Korea University Anam Hospital (Seoul, Korea) from a patient with infectious symptoms (IRB registration number:2020AN0157; 4 March 2021). CRAB C0 harbors *OXA-23* and is resistant to β-lactam antibiotics.

### 4.2. Materials

Rhamnetin (021136S), isorhamnetin (021120S), and tamarixetin (purity ≥ 99% by HPLC; 021140S) were purchased from the Indofine Chemical Company (Hillsborough, NJ, USA). Quercetin (purity ≥ 95% by HPLC; catalog number: Q4951) was purchased from Sigma-Aldrich (St. Louis, MO, USA). Flavonoids were dissolved in dimethyl sulfoxide at a concentration of 10 mg/mL and then diluted to be used in the appropriate medium for each experiment. LPS extracted from *E. coli* O111:B4 (L3024) was purchased from Sigma-Aldrich.

### 4.3. In Vitro Cytotoxicity Assay

RAW 264.7 murine macrophage cells and HEK cells were purchased from the Korea Cell Line Bank (Seoul, Korea). The cells were cultured in Dulbecco’s modified Eagle’s medium (DMEM; Welgene, Gyeongsan, Korea) supplemented with 10% fetal bovine serum and 1% penicillin/streptomycin and incubated in a humidified 5% CO_2_ incubator set at 37 °C. The cytotoxicity of the flavonoids was analyzed using a WST-8 cell proliferation kit (Biomax Co, Ltd., Seoul, Korea) according to the manufacturer’s instructions. Briefly, cells (1 × 10^5^) were seeded in 96-well plates, treated with flavonoids (0–50 μM) when they reached 80% confluence, and then incubated for 24 h. Then, a volume of WST-8 reagent equivalent to 10% of the volume of the total medium was added, and the absorbance at 450 nm was measured using a SpectraMAX microplate reader (Molecular Devices, San Jose, CA, USA). The values were expressed as a percentage of viability of control cells.

### 4.4. Infection of Macrophages with Bacteria

Bacteria were cultured in Luria Bertani (LB) medium until the optical density (OD) at 600 nm reached 0.6, centrifuged at 8000 rpm for 5 min, and suspended in DMEM. The bacterial suspension was used to infect the RAW 264.7 cells (1 × 10^5^) such that the final concentration of *E. coli* K1 was 1 × 10^6^ CFU and that of CRAB C0 was 5 × 10^5^ CFU.

### 4.5. Quantification of Inflammatory Markers in Murine Macrophages

RAW 264.7 cells (1 × 10^5^) were seeded in 96-well plates and treated with flavonoids and 20 ng/mL LPS for 1 h. After 16 h of incubation, NO production was quantified using the Griess reagent (Sigma Aldrich, St. Louis, USA). After adding the same amount of Griess reagent to the cell culture supernatant, the absorbance was measured at 540 nm. Nitrite content was evaluated using a standard curve of sodium nitrite. After treating the cells in the same way, the amounts of TNF-α and IL-6 present in the culture supernatant were determined using an enzyme-linked immunosorbent assay (ELISA) kit (R&D Systems, Minneapolis, MN, USA) according to the manufacturer’s protocol. The absorbance at 450 nm was measured using a SpectraMAX microplate reader (Molecular Devices, San Jose, CA, USA). TNF-α and IL-6 were quantified after creating a standard curve using the standard material included in the kit. All experiments were performed in triplicates.

### 4.6. Animals

All procedures were approved by the Institutional Animal Care and Use Committee (IACUC) of Konkuk University, Seoul, Korea (IACUC number: KU21204). Six-week-old female cancer research institute (ICR) mice were purchased from Orient (Daejeon, Korea). All mice were housed under specific pathogen-free conditions in an atmosphere of controlled temperature and humidity, and water and food were provided ad libitum.

### 4.7. Measurement of Antiseptic Activity of Rhamnetin in a Mouse Model of Septic Shock

*E. coli* K1 and CRAB C0 were used as the infectious strains. Control mice were injected with PBS (pH 7.4), and rhamnetin-treated mice were injected with 1 mg/kg rhamnetin. Mice infected with *E. coli* K1 (3 × 10^5^ CFU/mouse) and CRAB C0 (5 × 10^6^ CFU/mouse) were used as septic shock model controls. All injections were administered intraperitoneally. Mice were randomly assigned to three mice in each group. The mice were pretreated with 1 mg/kg rhamnetin for 1 h and then with bacteria, similar to that performed for mice with sepsis. After 16 h, the mice were sacrificed via cervical dislocation under ether-induced anesthesia. TNF-α and IL-6 levels in serum and lung lysates were quantified using a kit containing the antibody for each protein. (ELISA; R&D Systems). All procedures were performed in accordance with the manufacturer’s instructions.

### 4.8. Evaluation of Bacterial Clearance

After sacrificing the mice, the lungs, liver, and kidneys were aseptically removed and separately placed in sterile ice-cold PBS. After the tissues were blended using a homogenizer, the homogenate was diluted 1000-fold with PBS, and 10 μL of the diluted homogenate was plated on LB agar plates. The colonies were counted after incubation at 37 °C for 16 h.

### 4.9. Measurement of AST, ALT, and BUN Levels in Mouse Serum

AST, ALT, and BUN levels in the sera isolated from mice were detected using a kit provided by Asan Pharmaceutical (Hwaseong-si, South Korea) as described in previous study [36]. After the serum samples of each group were allowed to react with the substrate provided in the kit, absorbance was measured at 505 nm for AST and ALT measurements and at 580 nm for BUN measurement.

### 4.10. Histological Evaluation of Lung Tissue

The lung tissue was fixed with paraformaldehyde (4% *v*/*v*), prepared as a paraffin block, and sectioned to a thickness of 6 mm. After deparaffinization with xylene, rehydration was performed with gradient of ethanol concentrations and stained with hematoxylin and eosin. The lung tissue sections were then prepared on microscope slides and examined under a light microscope (Eclipse Ni; Nikon, Tokyo, Japan).

### 4.11. Statistical Analysis

All experiments were performed at least in triplicate, and the data are presented as the mean ± standard error of the mean (SEM) of independent experiments. One-way and two-way analyses of variance (ANOVA) followed by Dunnett’s tests were performed using the GraphPad Prism software (GraphPad Software Inc., La Jolla, CA, USA). Values were considered statistically significant at * *p* < 0.05, ** *p* < 0.01, and *** *p* < 0.001; ns indicates not significant.

## 5. Conclusions

The demand for drugs against infections caused by MDR gram-negative bacteria, especially CRAB, which is associated with a high risk of fatality, is increasing. In this study, we examined the potency of rhamnetin, a flavonoid with low cytotoxicity and high anti-inflammatory activity, for treating gram-negative bacterial infections. The decrease in pro-inflammatory cytokine production in the lung lysates of infected mice confirmed that rhamnetin enhances immunity by regulating cytokine secretion in the lungs of mice with gram-negative sepsis. In addition, microscopic observation of the morphological changes in the lung showed inhibition of lung inflammation and recovery from lung damage caused by CRAB-induced sepsis. Thus, rhamnetin presents a new breakthrough in the treatment of CRAB infection because of its anti-inflammatory, antibacterial, and pulmo-protective activities.

## Data Availability

The data presented in this study are available on request from the corresponding author.
